# Highly clarithromycin-resistant *Helicobacter pylori* infection in asymptomatic children from a rural community of Cajamarca-Peru

**DOI:** 10.1186/s13104-018-3919-z

**Published:** 2018-11-14

**Authors:** Miguel Angel Aguilar-Luis, Fernando Palacios-Cuervo, Fátima Espinal-Reyes, Andrea Calderón-Rivera, Saúl Levy-Blitchtein, Carlos Palomares-Reyes, Wilmer Silva-Caso, Victor Zavaleta-Gavidia, Jorge Bazán-Mayra, Angela Cornejo-Tapia, Juana del Valle-Mendoza, Luis J. del Valle

**Affiliations:** 1 School of Medicine, Research and Innovation Center of the Health Sciences Faculty, Universidad Peruana de Ciencias Aplicadas, Av. San marcos Cdra 2, Chorrillos, Lima, Peru; 2 Laboratorio de Biología Molecular, Instituto de Investigación Nutricional, Lima, Peru; 3 Dirección Regional de Salud de Cajamarca (DIRESA-Cajamarca), Cajamarca, Peru; 4 Barcelona Research Center for Multiscale Science and Engineering, Departament d’Enginyeria Química, EEBE, Universidad Politecnica de Catalunya (UPC), Barcelona Tech, Barcelona, Spain

**Keywords:** *Helicobacter pylori*, PCR, Clarithromycin, Gastric disease, Peptic ulcer disease

## Abstract

**Objective:**

The objective of this study was to determine the prevalence of clarithromycin-resistant *Helicobacter pylori* in asymptomatic children in a rural community of Cajamarca (northern Peru).

**Results:**

*Helicobacter pylori* was detected in 17.2% (49/285) of the samples. Unboiled water consumption the most frequent associated factor in patients with positive PCR for *H. pylori* infection (93.9%). Clarithromycin resistant mutations were found in 79.6% (39/49) of the positive samples for *H. pylori*. The most frequent mutation was A2142G (46.9%), followed by the double-mutation A2142G–A2143G (28.6%).

## Introduction

*Helicobacter pylori* is one of the most prevalent human pathogens and the major cause of chronic gastritis, peptic ulcer disease, gastric adenocarcinoma and mucosa-associated lymphoid tissue lymphoma in children and adults [1, 2]. More than half of the world’s population is infected, with approximately 4.4 billion individuals in 2015 [3]. Infection is related mainly to socioeconomic status and living conditions so higher prevalence rates (more than 40%) are predominantly seen in low or low-to-middle-income areas [4].

*Helicobacter pylori* infection is generally acquired during childhood and persists throughout life if untreated. The continuous exposure is associated with the presence of gastritis and other severe complications [2]. Virulence factors, host gastric mucosal factors and patient’s environment are associated with clinical outcomes [5].

No clinical manifestations and especially not recurrent abdominal pain, have been shown to be specific to *H. pylori* infection in children [2, 6, 7]. Both invasive and non-invasive methods have been described for the *H. pylori* detection [8]. Invasive tests require an endoscopic sample and include: histologic analysis, rapid urease test and cultures. On the other hand, the urea breath test, serologic tests and the antigen detection in feces are non-invasive methods with limited applications [9]. In that sense, a positive serologic result cannot differentiate between a current and a past infection and the urea breath test requires very sophisticated equipment that usually is not available in the low-income settings [9].

In children, the collection and analysis of stool samples can be of special interest, due to their easy and fast sample collection. However, few methods are used for *H. pylori* detection in feces, including bacterial cultures, polymerase chain reaction (PCR) and enzymatic immunoassays [10–12].

In 1992, a Peruvian study showed extremely high levels of by *H. pylori* infection in all natural regions of the country, with regions that registered a prevalence higher than 80% [13]. Most recently an analysis of 1711 patients diagnosed with *H. pylori* by 13C-urea breath test showed a prevalence of 45.6% in Lima, the capital of Peru [14]. Also a local study associated the first *H. pylori* infection in infancy with more diarrheal episodes and diminished linear growth [15].

In the last few decades, non-invasive tests have been gaining importance for the early detection of *H. pylori*, especially in the following of treated infants [4, 11, 16]. The *H. pylori* eradication can drastically reduce both gastric and duodenal ulcers rates in children, as well as prevent further complications.

Most of the current regimens for the eradication of *H. pylori* include clarithromycin as main antibiotic. However, there are differences in the prevalence of clarithromycin-resistant strains of *H. pylori* among different countries [10]. It is also very worrying that the incidence of clarithromycin-resistant strains of *H. pylori* in children is increasing worldwide [10]. *H. pylori* clarithromycin resistance is related to the structural change of 23S rRNA because it is the target of clarithromycin [10]. This structural change of 23S rRNA is caused by the single nucleotide polymorphism (SNP) of the 23S rRNA gene, at the position of 2142 or 2143 in most cases [10, 17].

The main objective of this study is to determine the frequency of children infected with *H. pylori*, the presence of clarithromycin resistance strains and determine associated factors for this infection in a rural community of Cajamarca.

## Main text

### Methods

#### Study area

San Pablo district is a rural area located at the Peruvian North in the Cajamarca department. San Pablo has a population of 13,591 habitants, most of them having a low socioeconomic status [18].

#### Patients and ethics

Children between 5 and 12 years old from the single Primary School of San Pablo district who had lived for at least the previous 6 months in the study area were considered in this study. Children who had received antiparasitic treatment or laxatives in the previous 15 days from sample collection were excluded from the study.

The study protocol was approved by the Ethics committee of the *Hospital Regional Docente* de Cajamarca and performed according to the Helsinki declaration. Informed consent was obtained from the parents or guardians of the children before sample collection. Stool samples were stored by the Regional Laboratory of the DIRESA Cajamarca as part of their parasitic surveillance system. Permits were also requested from DIRESA Cajamarca for the use of this samples.

#### Sample preparation

One stool sample was obtained from each participant, they were collected in sterile containers and stored at − 20 °C.

#### DNA extraction

DNA was extracted from above mentioned sample aliquots using a commercial kit (High Pure Template Preparation Kit, Roche Applied Science, Germany) according to the manufacturer instructions.

#### PCR amplification for detection of *H. pylori*

Presence of *H. pylori* was determined by PCR amplification of the *23S rRNA* gene and resistance mutation sequences for clarithromycin (A2142G and A2143G) were recognized by specific primers and conditions previously described [19].

#### Statistical analysis

The numbers of cases are given as frequencies and the graphics prepared with the Origin Pro software. Statistical difference in the frequency between the two different groups was assessed by normalized difference of two proportions test (*Z*-*test*) (Minitab^®^ 18 Statistical Software). All *p*-values were two-tail and *p *< 0.05 were considered statistically significant.

### Results

A cross-sectional study was conducted in the district of San Pablo in Cajamarca-Peru.

A total of 285 children from 6 to 14 years old were included in the study. None presented any suspicious symptom or diagnostic of *H. pylori*.

*Helicobacter pylori* was detected in 17.2% (49/285) of samples after PCR amplification and confirmed DNA sequencing. Demographic characteristics in the 285 children are summarized in Table 1. The population studied showed the same distribution by sex and the majority age group was in the range of 6–11 years. However, the frequency of male children infected (65.3%) with *H. pylori* was significantly higher (*p *= 0.013) than the cases of infected female children (34.7%). Both distribution of age and drainage systems in homes (e.g. bathroom and latrine) did not show an influence on the frequency of infected children (Table 1).

**Table 1:** Demographics characteristics in positives samples with diagnosis *H. pylori* by PCR from Cajamarca (Peru)

Characteristics	Diagnosed cases for *H. pylori* by PCR				
	Negatives		Positives		*p*
	n = 236	(%)	n = 49	(%)	(*Z*-test)
Gender					
Male	110	46.6	32	65.3	
Female	126	53.4	17	34.7	0.013
Age (years)					
6–11	223	94.5	42	85.7	
12–14	13	5.5	7	14.3	0.092
Sewage system at home					
Bathroom	71	30.1	12	24.5	
Latrine	165	69.9	37	75.5	0.413

**Fig. 1:**
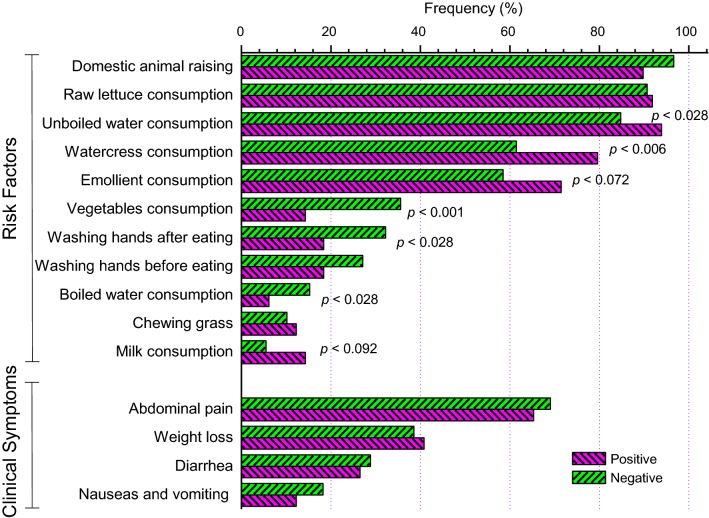
Risk factors and clinical symptoms during the last 3 months in the asymptomatic population of children

Clinical symptoms were evaluated as shown in Fig. 1. The abdominal pain (65.3%) was the most frequent symptom, followed by weight loss (40.8%), diarrhea (26.5%), and nausea-vomiting (12.2%) in last 3 months in patients with positive PCR for *H. pylori* infection. However, the frequencies of these symptoms did not show significant differences with the cases of non-infected children (Fig. 1).

On the other hand, factors associated with *H. pylori* infection were evaluated as shown in Fig. 1. Unboiled water (93.9%) and watercress (79.6%) consumptions were the most significant factors associated with H. pylori infection (with p = 0.028 and p = 0.006, respectively) in infected children compared to children who were not infected.

**Fig. 2:**
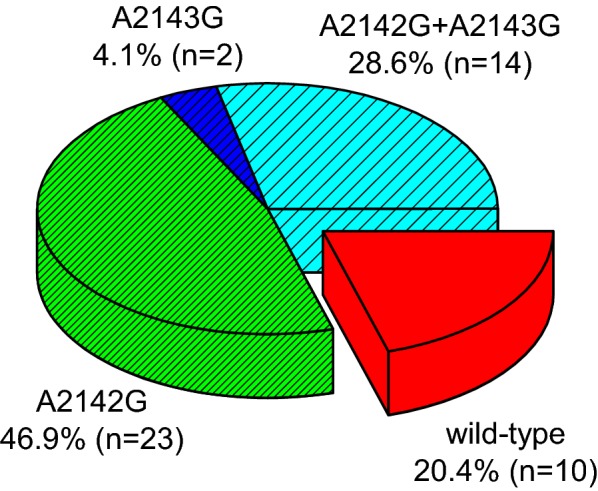
Frequency of clarithromycin-resistant mutations in the 23S rRNA gene of *H. pylori* in the children population

In addition, other habits such as vegetable consumption, hand washing before eating and boiled water consumption could favor non-infection by *H. pylori* (Fig. 1). In the samples that were positive for the presence of the *H. pylori* bacterium, the target gene (23S rRNA) sequence for the antibiotic clarithromycin was studied. Point mutations from adenine (A) to guanine (G) in the nucleotide 2142 or 2143 of this gene produce resistance to clarithromycin. A total of 79.6% (39/49) of the positive cases for *H. pylori* showed some clarithromycin-resistant mutation while 20.4% (10/49) of positive cases did not present mutations in this gene (wild-type in Fig. 2). The occurrence of single mutations (A2142G or A2143G) in children with clarithromycin-resistant *H. pylori* infection 51% (23/49) was significantly higher compared with the occurrence of double mutations (A2142G-2143G) 28.6% (14/49); (p = 0.02).

### Discussion

We observed a prevalence of 17.2% for *H. pylori* in our pediatric population, which is lower than previous reports in Peru [13, 14]. However, in this study we observed that the presence of the *H. pylori* bacterium is not clearly related to a clinical diagnosis of the infection (Fig. 1). In addition, in our population the 85% of cases with the presence of *H. pylori* are found in children with ages in the range of 6–11 years (Table 1), and it could be suggesting that infection may be age-dependent [20]. Although a previous study in Peru demonstrated a risk of early *H. pylori* infection in infants [21]. Other studies in Latin America, such as those conducted in Brazil, show a decrease in the prevalence of *H. pylori* infection in a period of 10 years in children under 18 years of age [22].

Recently, emphasis has been placed on the use of non-invasive diagnostic methods for the detection of *H. pylori* in pediatric population [23, 24]; however, their use is not yet standardized in routine clinical practice. The PCR is among the most promising techniques for being faster, more accurate and specificity [16, 25]. This method can detect *H. pylori* regardless of their viability with high sensitivity and specificity arriving to 95% [26]. PCR presents greater specificity than the rapid urease test (RUT) [25]. In addition, PCR has the advantage of identifying *H. pylori* with a limited quantity of bacterial load allowing to recognize the infection when other tests give negative results [27]. Thus, Montealegre et al. reported a prevalence of *H. pylori* of 78.3% using histological diagnosis and 68.3% by PCR [28]. In another study, ELISA detection of *H. pylori* antigen in stool samples was compared against other invasive methods, demonstrating the presence of the antigen with a 90% sensitivity and 100% specificity; and a positive predictive value of 100% [29]. In 2012, Smith et al. conducted a study in in dyspeptic patients comparing the detection of *H. pylori* by urease breath test (UBT) and PCR in stool samples, in this study a prevalence of 20.6% was reported and a specificity of 100% was calculated for the PCR when compared to the UBT as a gold standard [30].

Our study demonstrates the advantages of using the PCR as a method to detect the presence of *H. pylori* in stool samples. We found a prevalence of 17.2% and only found one study in Peru that used PCR to detect *H. pylori* in stool samples [31], while most of studies in the country focused on other methods as fast UBT [32].

For *H. pylori* eradication clarithromycin-based triple therapy has been recommended in children and adults by Maastricht Consensus, however the prevalence of resistant strains was higher in children and the frequent point mutations that are responsible for the clarithromycin resistance included A2143G, A2142G, A2142C and A2144G, and they varied geographically [10]. Rapid, reliable and non-invasive methods for detecting clarithromycin resistant *H. pylori* are needed [8, 10]. In this study clarithromycin resistant mutations were found in 79.6% (39/49) of the samples, which confirms the high prevalence in children. Most of the samples had one (51%) or two mutations (28.6%). The simple mutation most frequent was A2142G (46.9%), followed by A2143G (4.1%) (Fig. 2). However, the opposite happens in other reports for these mutations [10], which could suggest that these differences are due to different clones or simply that the bacteria are subjected to different environmental stresses that favors one or the other mutation; in any case, this requires further studies.

Since macrolide antibiotics show cross resistance, the higher clarithromycin resistance rates could be attributed to indiscriminate use of erythromycin and azithromycin in our population for respiratory infections [33]. Antibiotic resistance is an important conditioning factor for the success of *H. pylori* treatment. For this reason it is recommended in populations with a high rate of resistance to perform antibiotic sensitivity tests for clarithromycin before implementing the triple therapy [34].

The high prevalence of *H. pylori* in asymptomatic persons remains a special concern in our settings, considering the carcinogenic potential of *H. pylori* and the high prevalence of gastric cancer in Peru, which is estimated to be present in 15.8 per 100.000 inhabitants [35].

## Limitations

The range of detection rates reported with molecular methods compared to conventional tests is very wide. This may be due to the fast degradation of the bacteria in the gastrointestinal tract which leads to a very low concentration of the *H. pylori* in the feces [11, 12]. This is a study in a small rural community in Peru and the results may differ from the reality of urban areas.

## Abbreviations

PCR: polymerase chain reaction
DNA: deoxibonucleic acid
bp: base pairs
*H. Pylori*: *Helicobacter pylori*
rRNA: RNA ribosomal ribonucleic acid
bp: base pairs
